# Multimedia versus audio-based education for improving dentists’ nutrition-related knowledge and behavior: A randomized comparative study

**DOI:** 10.1371/journal.pone.0360426

**Published:** 2026-10-28

**Authors:** Mehdi Farhadi, Afsaneh Pakdaman, Mahdia Gholami

**Affiliations:** 1 Department of Prosthodontics, School of Dentistry, Tehran University of Medical Sciences, Tehran, Iran; 2 Research Center for Caries Prevention, Dentistry Research Institute, Tehran University of Medical Sciences, Tehran, Iran; 3 Department of Community Oral Health, School of Dentistry, Tehran University of Medical Sciences, Tehran, Iran; The University of Oklahoma College of Dentistry, UNITED STATES OF AMERICA

## Abstract

**Background:**

Nutrition plays a crucial role in oral health, influencing the risk of caries, periodontal disease, erosion, and oral cancers. Dentists are key providers of dietary guidance, but require adequate knowledge to do this effectively. This study compared the impact of multimedia versus audio-based educational interventions on the knowledge and self-reported behavior of general dentists in Tehran regarding nutrition and oral health.

**Methods:**

In this randomized controlled trial, 166 general dentists in Tehran were recruited in 2023 through professional online platforms and randomly allocated to either a multimedia-based or an audio-based educational intervention. Knowledge (11 items) and behavior (9 items) regarding the impact of nutrition on periodontal health, dental caries, oral cancer, and erosion were assessed using a validated questionnaire. The educational intervention was delivered over a one-month period. Within-group changes were assessed using paired t-tests, between-group differences using repeated-measures ANOVA, and the effect of demographic variables on post-intervention scores using ANCOVA, with baseline values as covariates (α = 0.05).

**Results:**

Of the participants, 53.6% were women, and 37.9% worked in private practice. Both groups demonstrated significant improvements in knowledge and behavior scores following the intervention (p < 0.001), with greater gains observed in the multimedia group. Analysis of covariance indicated that baseline knowledge and behavior were not significantly associated with age, gender, dental school, years of professional experience, or practice setting.

**Conclusion:**

Both multimedia- and audio-based educational interventions effectively improved dentists’ knowledge and self-reported behaviors related to nutrition and oral health. Therefore, given dentists’ busy work schedules, the use of these educational approaches may enhance their capacity to provide evidence-based counseling in the field of nutrition and oral health.

## Introduction

Nutrition plays a decisive role in determining oral health status across the lifespan. Adequate intake of macronutrients and micronutrients supports the development and maintenance of oral tissues, while nutritional deficiencies contribute to a wide spectrum of oral diseases, including dental caries, periodontal diseases, mucosal lesions, and salivary dysfunction. A balanced diet has been recognized as a determinant of general health and oral health, influencing biological, behavioral, and immunological processes that shape susceptibility to oral conditions [1–3]. The interdependence between systemic nutrition and oral structures extends beyond physiological pathways; compromised oral health may also restrict dietary choices, thereby creating a bidirectional association that reinforces the need for preventive strategies [4,5].

Diet-related risk factors significantly contribute to the global burden of oral diseases. High consumption of fermentable carbohydrates is a well-established cause of dental caries, especially when combined with poor oral hygiene and frequent intake [6–9]. Similarly, inadequate intake of essential vitamins and minerals may impair periodontal integrity, reduce immune function, and hinder wound healing [2,10,11]. Nutritional imbalances also influence the risk of oral cancer, with evidence linking carcinogenic dietary patterns and habits—such as areca nut use and low fruit and vegetable consumption—to malignant transformation in the oral mucosa [12,13].

These findings underscore the essential role of nutritional counseling in oral health care. Dentists are uniquely positioned to provide dietary advice aimed at preventing or mitigating oral diseases. Their routine interactions with patients offer an opportunity to integrate tailored nutrition counseling into clinical practice. However, existing evidence indicates that dental professionals often have insufficient knowledge of nutrition and its relevance to oral health [14–18].

Studies conducted among dental students and practitioners in various contexts, including Iran, highlight gaps in awareness regarding dietary risk factors, preventive strategies, and evidence-based nutritional recommendations [19]. Such deficiencies may limit dentists’ capacity to guide patients effectively, thereby reducing the potential preventive impact of dental visits. Continuing professional education provides a pathway to address these knowledge gaps. Advances in digital technologies have facilitated diverse educational methods, including multimedia-based learning and audio-based instruction [20]. Multimedia interventions combine visual, auditory, and interactive components, which may enhance engagement and comprehension by appealing to multiple learning modalities. Audio-based methods, on the other hand, offer flexibility and accessibility, enabling learners to receive educational content in a convenient format [21,22].

Previous research has demonstrated that structured educational interventions can improve oral-health-related knowledge and promote healthy behaviors among various populations [23]. However, to our knowledge, no study has directly compared the effectiveness of multimedia-based versus audio-based education formats in improving dentists’ nutrition-related knowledge and behavior. Given the central role of nutrition in oral disease prevention and the need for evidence-based dietary counseling in dental practice, identifying which educational modality most effectively enhances dentists’ competence in this area remains an important, unaddressed question [19].

This study was therefore designed to compare the effectiveness of two forms of digital education—multimedia-based and audio-based—on the knowledge and self-reported behavior of general dentists in Tehran regarding nutrition and oral health. Based on multimedia learning theory, which posits that combining visual and auditory information channels enhances information process and retention compared with a single-channel format [24], we hypothesized that the multimedia-based intervention would produce greater improvements in knowledge and behavior than the audio-based intervention, which delivers identical content through a single channel.

## Methods

### Study design

This study was designed as a randomized controlled educational intervention conducted in 2023 among general dentists practicing in Tehran. The objective was to compare the effectiveness of two instructional modalities—multimedia-based and audio-based—on dentists’ knowledge and self-reported behavior regarding nutrition and oral health. The research followed a pre–post intervention format in which participants completed validated questionnaires before and after the one-month educational period (between August 6, 2023, and September 6, 2023). All stages of the study adhered to the ethical and academic standards outlined by the Tehran University of Medical Sciences Ethics Committee. Participant flow diagram through the study is presented in Fig 1.

**Fig 1:**
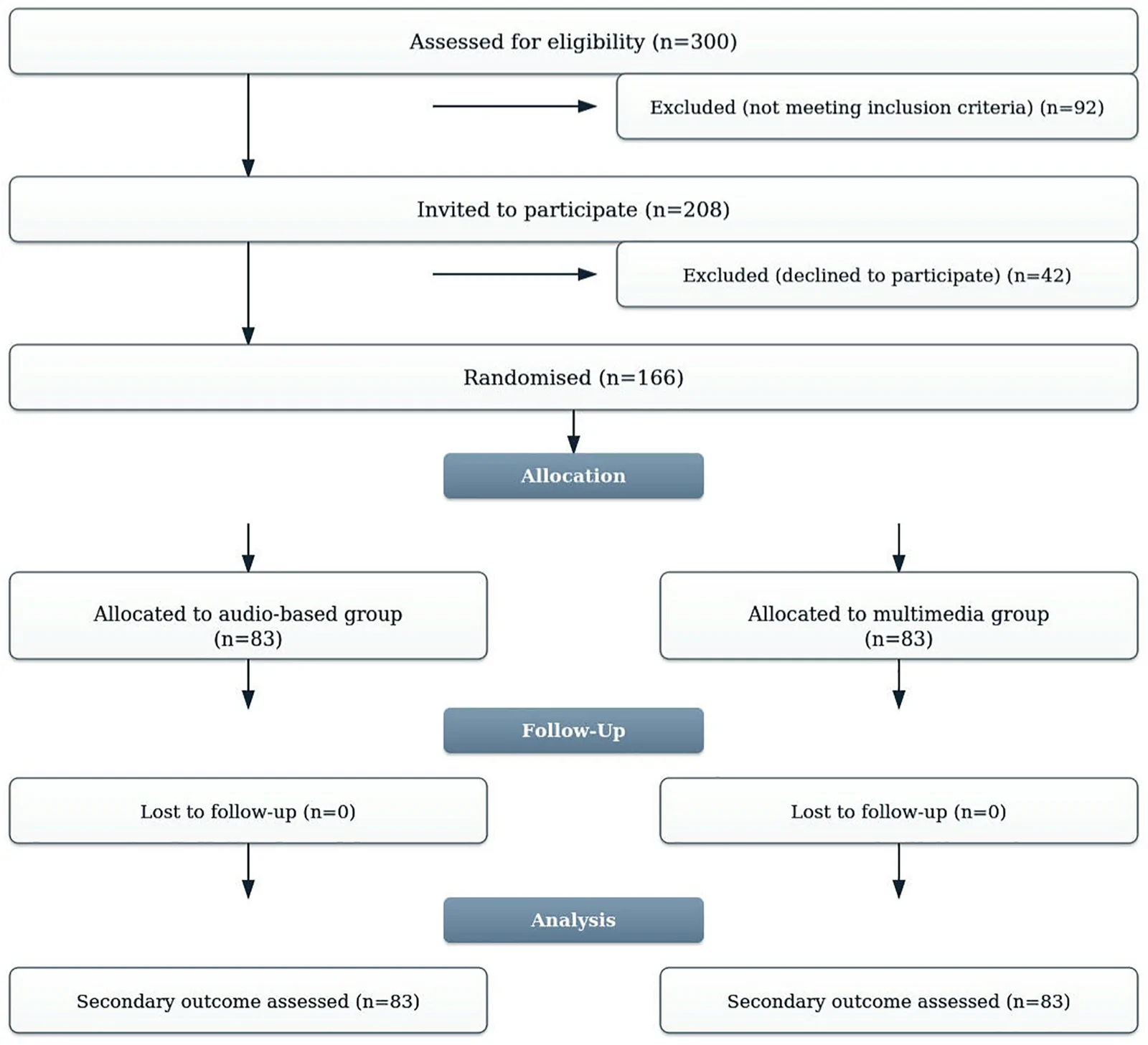
Flow diagram of study participant.

### Participants and recruitment

Participants were general dentists working in Tehran who were accessible through professional and educational groups on social media platforms, including Telegram, WhatsApp, and Bale. A total of 166 dentists were recruited through convenience sampling. The inclusion criteria were: (1) holding a degree in general dentistry, (2) currently practicing within Tehran, (3) willingness to participate in both phases of the study, and (4) access to a digital device capable of receiving multimedia or audio educational content. Exclusion criteria consisted of incomplete questionnaire responses or failure to engage with the assigned educational materials during the intervention period.

After confirming eligibility and obtaining informed electronic consent, participants were randomly assigned to one of two groups using a simple random allocation procedure. They were randomly allocated to the multimedia or audio-based intervention group with a 1:1 allocation ratio. Randomization was performed by writing each participant’s name on a separate piece of paper and drawing lots to determine group assignment. This procedure was conducted by one of the investigators, who was also responsible for communicating with participants and distributing the questionnaires and interventions to each group. Both groups received the same subject matter, differing only in the modality of content delivery.

### Study instruments

Data were collected using an online questionnaire via Porsline designed by the researchers and evaluated for validity and reliability. The use and processing of the collected data were conducted in accordance with the platform’s applicable Terms of Service and relevant rules and regulations. To identify appropriate items for the questionnaire, a comprehensive review of existing related literature and instruments was conducted. The initial version of the questionnaire was independently revised by two experts. Face and content validity of the questionnaire items were then assessed. Face validity was evaluated using a qualitative approach, focusing on the appropriateness of the items’ appearance as well as their clarity and comprehensibility from the perspective of the expert reviewers and ten general dentists.

Content validity was reviewed by a 10-member expert panel (8 specialists in community oral health and 2 in nutrition), who rated each item’s relevance, clarity, simplicity, and necessity to calculate the Content Validity Index (CVI) and the Content Validity Ratio (CVR). Item-level CVI (I-CVI) ranged from 0.7 to 1, with a scale-level CVI (S-CVI/Ave) of 0.88, CVR values ranged from 0.6 to 1, with a mean CVR of 0.84. Items were retained, revised, or removed based on the acceptable thresholds of CVI (0.7) and CVR (60%) [25,26].

Test-retest reliability was assessed in a pilot sample of 10 participants who completed the questionnaire twice at a 10-day interval; the intraclass correlation coefficient (ICC) was calculated for the total knowledge and behavior domain scores (interpreted as poor <0.40, good 0.40–0.75, and very good >0.75). The ICC values for the knowledge and behavior scores were 0.656 and 0.617, respectively (both in the “good” range). Internal consistency was assessed using Cronbach’s alpha, with a value above 0.70 considered an acceptable threshold; the overall Cronbach’s alpha coefficient across all questionnaire items was 0.780 [27,28].

As a result of this process, the final version of the instrument comprised three sections. The first section collected demographic information, including participants’ age, sex, university of graduation, years since graduation, and primary workplace setting (public clinic, private office, or other). The second section consisted of an 11-item knowledge assessment that evaluated participants’ understanding of the relationship between diet and oral health, including dietary factors associated with dental caries, periodontal health, nutritional deficiencies, and dietary risk factors for oral cancer. The third section included a 9-item behavior assessment that evaluated self-reported professional practices related to nutrition counseling and patient education, such as conducting dietary assessments, providing preventive guidance on sugar consumption, and counseling patients about erosive and cariogenic foods. Sample Size

The sample size was calculated as 166 dentists. This was sufficient to detect group differences in score changes with appropriate statistical power based on expected moderate effect sizes commonly observed in educational intervention research [14]. Sample size was calculated using PASS version 11 software (Repeated Measures ANOVA Power Analysis option), based on the effect-size assumptions reported in a prior study on nutrition-related oral health knowledge and practice [14], with α = 0.05. A sample size of 83 dentists per group provided 80.3% power to detect the between-group (intervention type) effect (Sigma = 0.87) and 99.9% power to detect the within-group, pre-post effect (Sigma = 0.5).

### Data collection

Data were collected in two phases of Pre- and post-intervention. To assess dentists’ baseline knowledge and behavior, participants received a link to the online questionnaire and completed it before receiving any educational materials.

The educational intervention was designed in two formats: multimedia-based and audio-based. Each arm received a single educational file -one narrated video (multimedia arm) or one narrated audio recording (audio arm). The multimedia content combined visual and auditory elements (narrated videos), whereas the audio-based content consisted solely of narrated audio recordings. Both formats covered identical content, with approximately five minutes in length, focusing on key aspects of nutrition and oral health. Topics included the relationship between healthy nutrition and periodontal health, the role of sugar consumption in caries formation, the prevention of dental caries, oral cancer, and tooth wear, as well as the effects of cariostatic and anticariogenic foods [29–31].

Participants were randomly assigned to the multimedia or audio groups. Educational materials were delivered over one month through the WhatsApp and Telegram and Bale platforms. To reinforce engagement and ensure content delivery, participants received two to three text message reminders during the intervention. Engagement was monitored via participants’ report, supported by the reminder messages described above. Afterwards, the same questionnaire was re-administered to all participants to assess changes in knowledge and behavior. All participants who completed the baseline questionnaire also completed the post-intervention questionnaire; therefore, the final dataset contained no missing values.

### Ethical considerations

This study was approved by the Ethics Committee of Tehran University of Medical Sciences under the approval code IR.TUMS.DENTISTRY.REC.1401.153. Participation was voluntary, and all individuals provided informed consent. Confidentiality was strictly maintained; no identifying information was linked to questionnaire responses. Participants were informed that they could withdraw at any time. The study complied with all institutional requirements regarding academic integrity and research ethics.

### Statistical analysis

Data were analyzed using SPSS version 24. The data analyst was blinded during the analysis phase. Responses to knowledge questions were scored dichotomously as correct or incorrect, generating a total score ranging from 0 to 11. Responses to behavior questions received scores of 1, 0.5 and 0, with 1 being assigned to the most appropriate response. The total score was calculated based on the sum of the response scores, ranging from 0 to 9. Descriptive statistics were calculated for demographic variables and baseline scores. To assess the effectiveness of the interventions, paired t-tests were used to compare pre- and post-intervention scores within each group Changes in knowledge and behavior scores in the two groups were compared using Repeated Measure ANOVA, with the type of educational intervention as a between subject factor. Chi-square tests were used for categorical demographic comparisons. Analysis of covariance (ANCOVA) assessed the potential influence of demographic variables on post-intervention knowledge and behavior scores, adjusting for baseline values. The significance threshold was set at p < 0.05.

## Results

### Participant characteristics

A total of 166 general dentists participated in the study and completed both phases of data collection. Among these participants, 53.6% were women, and the remaining were men. The mean age of the participants was 32.5 ± 7.50 years. Most dentists had graduated between one and five years prior to the study; specifically, 46.4% reported 1–5 years of professional experience. Regarding workplace settings, 37.9% were engaged in private practice, whereas others worked in clinics, institutions, or mixed professional environments. Demographic characteristics of participants are presented in Table 1. As shown in Table 1, the audio-based and multimedia groups did not differ significantly in gender (p = 0.534) or years since graduation (p = 0.432), but did differ significantly in dental school type (p < 0.001) and workplace (p = 0.001).

**Table 1:** Demographic characteristics of general dentists participating in the study (N = 166).

Variable	Audio-based Group (N = 83) N(%)	Multimedia Group (N = 83) N(%)	p-value*
**Gender**			0.534
Female	47 (56.6)	42 (50.6)	
Male	36 (43.4)	41 (49.4)	
**Dental School Type**			<0.001
1	24 (28.9)	48 (57.8)	
2	56 (67.5)	31 (37.4)	
3	3 (3.6)	4 (4.8)	
**Elapsed Time Since Graduation**			0.432
1–5 years	35 (42.2)	42 (50.6)	
6–10 years	21 (25.3)	21 (25.3)	
> 10 years	27 (32.5)	20 (24.1)	
**Workplace**			0.001
Office	37 (44.6)	26 (31.3)	
Public Clinic	15 (18.1)	18 (21.7)	
Private Clinic	22 (26.5)	20 (24.1)	
Hospital	2 (2.4)	2 (2.4)	
Other	7 (8.4)	17 (20.5)	

*p < 0.05.

Table 2 shows the frequency of participants’ correct answer to knowledge questions before and after the intervention in two groups. Table 3 demonstrates the frequency of participants’ most appropriate answer to behavior questions before and after the intervention. Participants demonstrated better recognition of dietary risk factors for dental caries, improved understanding of micronutrient roles in periodontal health, and stronger alignment with recommended behaviors for nutrition-related patient counseling.

**Table 2:** Frequency of participants’ correct answers to knowledge questions related to nutrition and oral health before and after the intervention.

Question (*correct answer*)	Audio-based (Total = 83) N(%)		Multimedia (Total = 83) N(%)	
	Before	After	Before	After
1. Which of the following is not an effect of consuming crunchy and fibrous foods? (*Removes microbial plaque from the gingival margin*)	36 (43.4)	41 (49.4)	29 (34.9)	64 (77.1)
2. Which statement regarding dental erosion is correct? (*Consuming acidic foods before sleep causes the most damage*)	70 (84.3)	75 (90.3)	63 (75.9)	76 (91.5)
3. Which vitamin or nutrient does not reduce the risk of oral cancer? (*Vitamin K*)	41 (49.4)	53 (63.9)	38 (45.8)	56 (67.5)
4. Which of the following is a characteristic of cariostatic substances? (*Do not play a role in the initiation or progression of enamel demineralization*)	34 (41)	73 (87.9)	25 (30.1)	64 (77.1)
5. Which of the following is not considered cariostatic? (*Refined grains*)	34 (41)	41 (49.4)	29 (34.9)	41 (49.4)
6. Which type of sugar has the highest cariogenic potential? (*Sucrose*)	67 (80.7)	73 (87.9)	64 (77.1)	76 (91.6)
7. Which of the following is a property of anticariogenic substances? (*Neutralize cariogenic effects if consumed with cariogenic foods*)	70 (84.3)	74 (89.1)	49 (59)	74 (89.1)
8. Which of the following foods has anticariogenic properties? (*Cheese*)	69 (83.1)	70 (84.3)	59 (71.1)	71 (85.5)
9. What is the best time to consume sugar-containing foods? (W*ith the main meals*)	75 (90.3)	77 (92.7)	76 (91.5)	78 (93.9)
10. Which of the following statements about carbohydrate cariogenicity is incorrect? (*Natural sugars like honey have low cariogenicity*)	39 (47)	53 (63.9)	51 (61.4)	70 (84.3)
11. What is the optimal time to brush teeth after consuming a main meal? (*30 minutes after eating*)	68 (81.9)	70 (84.3)	52 (62.7)	72 (86.7)

**Table 3:** Frequency of participants’ appropriate answer to behavior questions related to nutrition and oral health before and after the intervention.

Behavior Question (*most appropriate answer*)	Audio-based (N = 83) N(%)		Multimedia (N = 83) N(%)	
	Before	After	Before	After
1. Do you consume foods from all groups according to the food pyramid? (*Always or most of the time*)	18 (21.7)	29 (34.9)	17 (20.5)	40 (48.2)
2. If you consume foods from all groups, do you consume them in adequate amounts? (*Always or most of the time*)	11 (13.3)	14 (16.9)	15 (18.1)	26 (31.3)
3. How many times a day do you consume sugar-containing foods? (*2–3 times*)	26 (31.3)	46 (55.4)	29 (34.9)	54 (65.1)
4. Do you drink water or rinse your mouth with water after consuming sugar-containing foods? (*Always or most of the time*)	5 (6)	10 (12)	13 (15.7)	19 (22.9)
5. Do you consume dairy products, such as milk or cheese, at the same time or immediately after eating sweets? (*Always or most of the time*)	0 (0)	0 (0)	4 (4.8)	4 (4.8)
6. Do you chew sugar-free gum after consuming sugary foods? (*Always or most of the time*)	1 (1.2)	1 (1.2)	3 (3.6)	4 (4.8)
7. Do you drink water instead of sweetened or carbonated beverages? (*Always or most of the time*)	5 (6)	6 (7.2)	15 (18.1)	21 (25.3)
8. Which of the following food groups do you eat most often as a snack? (*Nuts and seeds*)	37 (44.6)	40 (48.2)	23 (27.7)	21 (25.3)
9. On average, how many glasses of water do you drink daily? (*8 glasses or more*)	4 (4.8)	7 (8.4)	9 (10.8)	17 (20.5)

Fig 2 presents the mean total knowledge scores of the audio-based and multimedia groups before and after the intervention. Before the intervention, the audio-based group had a mean knowledge score of 7.52 ± 1.32, with scores ranging from 4 to 11, while the multimedia group showed a mean score of 6.51 ± 1.68, with values between 2 and 10. After the intervention, knowledge scores increased in both groups. The mean score rose to 8.95 ± 1.01 in the audio-based group and to 9.20 ± 1.28 in the multimedia group, with score ranges of 7–11 and 4–11, respectively.

**Fig 2:**
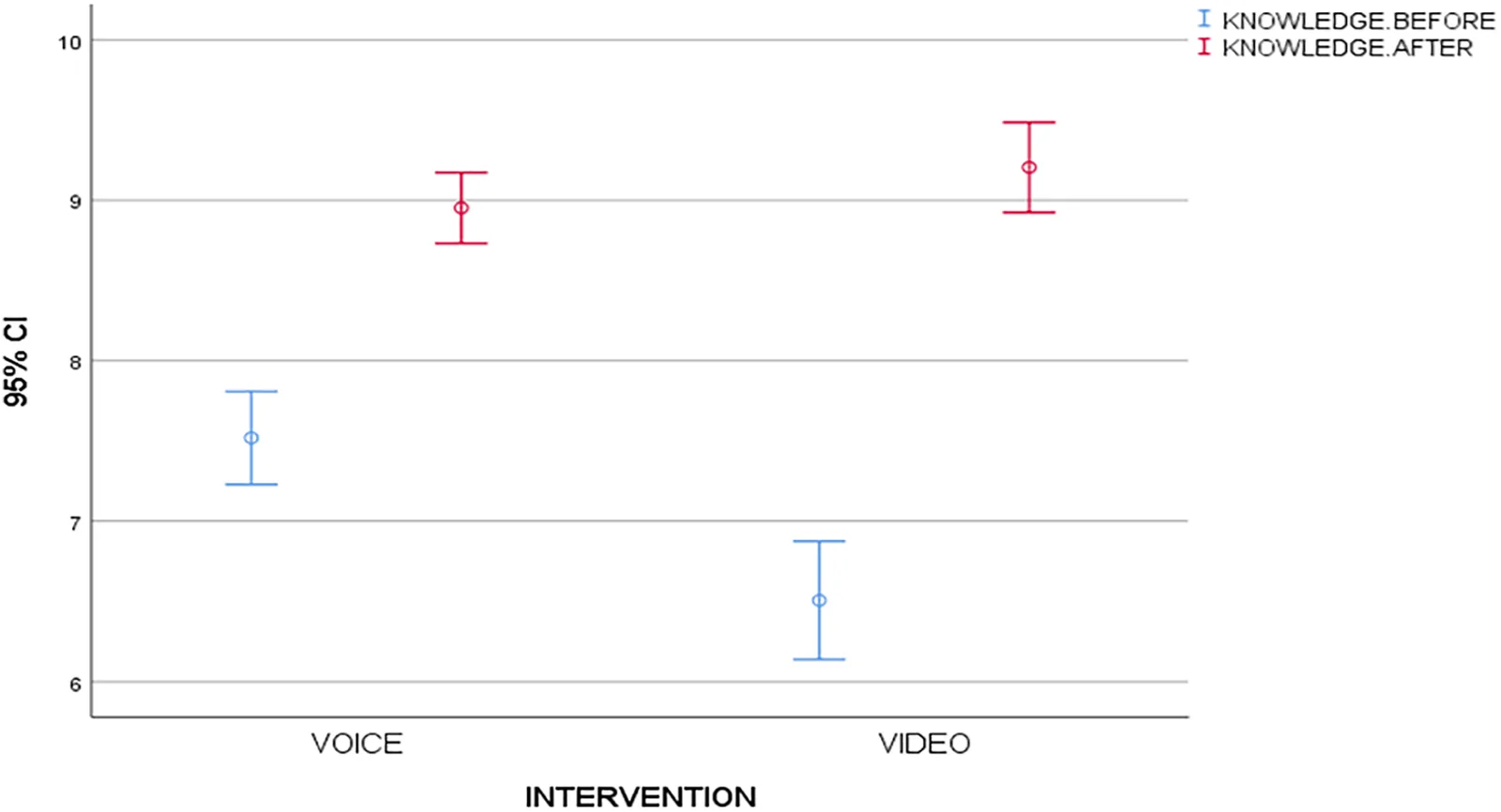
Comparison of the mean knowledge scores of participants on nutrition- and oral health–related questions before and after the intervention.

Fig 3 illustrates the mean behavior scores of the two groups before and after the intervention. Before the intervention, the mean behavior score was 3.90 ± 1.04 in the audio-based group and 4.00 ± 0.99 in the multimedia group, with scores ranging from 2 to 6.5. After the intervention, behavior scores improved in both groups, reaching a mean of 4.56 ± 1.08 in the audio-based group and 5.07 ± 0.99 in the multimedia group, with observed ranges of 2–7.5 and 3–8, respectively.

**Fig 3:**
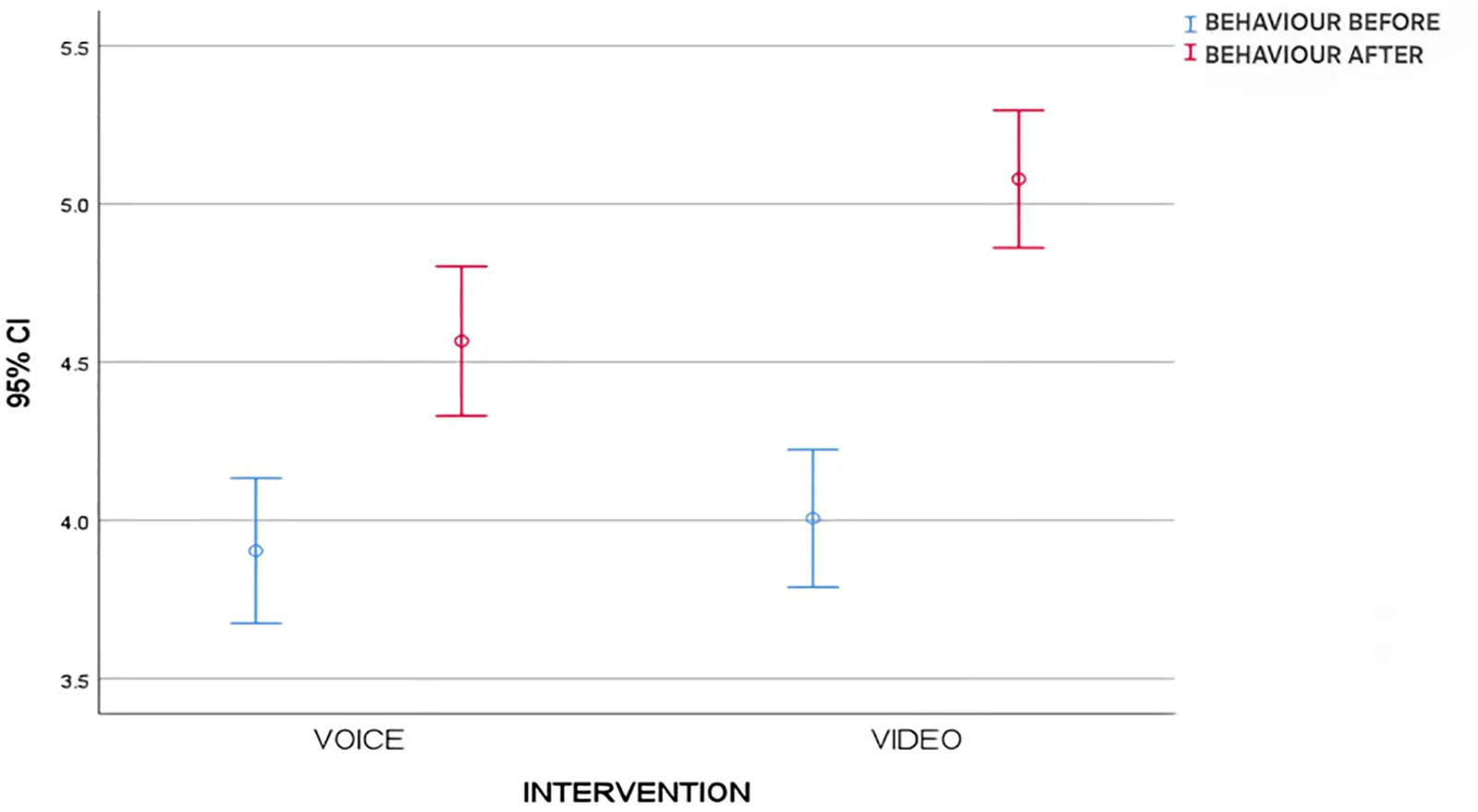
Comparison of the mean behavior scores of participants on nutrition- and oral health–related questions before and after the intervention.

The average increase in knowledge score was 1.43 ± 0.9 in the audio-based group and 2.70 ± 1.7 in the multimedia group. Statistical analysis using the t-test indicated that improvements in nutrition- and oral health–related knowledge were significant in both groups (p < 0.001; Cohen’s d = 1.55 for the audio-based group and 1.54 for the multimedia group); however, the increase observed in the multimedia group was greater, corresponding to a large between-group effect size (Cohen’s d = 0.96). The mean change in behavior score was 0.66 ± 0.55 in the audio-based group and 1.1 ± 0.8 in the multimedia group. The results of the t-test confirmed that the behavior improvements related to nutrition and oral health were statistically significant in both groups (p < 0.001; Cohen’s d = 1.20 for the audio-based group and 1.31 for the multimedia group), with a greater improvement observed in the multimedia group, corresponding to a small-to-moderate between-group effect size (Cohen’s d = 0.45) (Table 4).

**Table 4:** Changes in knowledge and behavior scores before and after the educational interventions.

Outcome	Intervention Group	Before, Mean ± SD	After, Mean ± SD	Mean change(95% CI*)	T (df)	Cohen’s d	P value**
**Knowledge**	Audio-based (n = 83)	7.52 ± 1.32	8.95 ± 1.01	+1.43(1.23 to 1.64)	14.10 (82)	1.55	<0.001
	Multimedia (n = 83)	6.51 ± 1.68	9.20 ± 1.28	+2.70(2.32 to 3.08)	14.04 (82)	1.54	<0.001
**Behavior**	Audio-based (n = 83)	3.90 ± 1.04	4.56 ± 1.08	+0.66(0.54 to 0.78)	10.92 (82)	1.20	<0.001
	Multimedia (n = 83)	4.00 ± 0.99	5.07 ± 0.99	+1.07(0.89 to 1.25)	11.92 (82)	1.31	<0.001

*Confidence Interval.

**P < 0.05.

Mixed repeated-measures ANOVA showed a significant main effect of time (F(1,164)=361.13, P < 0.001, partial η^2^ = 0.688), a significant main effect of intervention group (F(1,164)=4.50, P = 0.035, partial η^2^ = 0.027), and a significant time × intervention interaction (F(1,164)=33.84, P < 0.001, partial η^2^ = 0.171), indicating that the increase in knowledge scores differed significantly between the two educational interventions. ANCOVA results demonstrated that none of the demographic variables—including age, gender, academic background, professional experience, or workplace had a statistically significant influence on post-intervention scores after adjusting for baseline values.

## Discussion

The present study aimed to investigate the effectiveness of audio-based and multimedia-based educational interventions on general dentists’ knowledge and behavior concerning the relationship between nutrition and oral health. This study was conducted in Tehran in 2023. The results demonstrated that following the implementation of the educational interventions, both knowledge and behavior scores related to nutrition and oral health improved significantly in the audio-based and multimedia-based groups.

Comparative analyses of pre-intervention and post-intervention scores revealed that, although both groups exhibited statistically significant improvements in knowledge related to nutrition and oral health, the magnitude of change differed between the two intervention modalities. Specifically, the multimedia-based intervention resulted in a significantly greater increase in knowledge scores compared to the audio-based intervention. Similarly, evaluation of behavior outcomes indicated a statistically significant improvement in mean behavior scores related to nutrition and oral health in both groups after the intervention. These findings indicate that multimedia content—likely due to its combination of visual, auditory, and structured didactic elements—facilitated deeper comprehension and better recall compared with the audio-only format. Although both methods were effective, the magnitude of improvement was significantly greater in the multimedia group. A plausible explanation for this difference is offered by the cognitive theory of multimedia learning, which holds that people learn more effectively from words and pictures presented together than from words alone, because visual and auditory information are processed through separate cognitive channels and can be integrated to support deeper encoding and retrieval. By engaging both channels simultaneously, the multimedia format used in this study may have reduced cognitive load and facilitated better comprehension and recall of nutrition-related content than the audio-only format, consistent with findings from other digitally delivered educational interventions in dental and health-professional education [20,21].

However, the degree of improvement observed in the multimedia group exceeded that of the audio-based group, suggesting a superior effect of multimedia education on behavior -related outcomes.

Furthermore, the findings of the present study indicated no statistically significant associations between dentists’ knowledge and behavior scores related to nutrition and oral health and demographic variables, including age, sex, and time elapsed since graduation, at the pre-intervention stage. These findings are in contrast to those reported by Parveen, who examined dentists’ knowledge related to nutrition and oral health and found higher knowledge levels among older dentists, those with greater professional experience, and female dentists [32]. This difference may be explained by variations in the study populations, as the present study included dentists practicing within a single city, whereas Parveen’s study evaluated dentists from across the country. These findings of the present study are consistent with those reported by Meybodi, who similarly identified no significant relationships with these variables, except for a significant association between nutritional behavior and professional experience [15]. The observed discrepancy regarding work experience may be attributable to differences in categorization methods, as Meybodi classified dentists into two groups based on a five-year threshold, whereas the present study employed a three-tier classification of 1–5 years, 6–10 years, and more than 10 years of professional experience. In addition, the results of the present study were in agreement with the findings of Chalmuri Y et al., who reported no significant association between gender and nutritional knowledge among dental students [33]. In contrast, these findings differ from those reported by Badrasawi et al., who demonstrated higher levels of nutritional awareness among women compared to men [34]. This inconsistency may be explained by differences in the target populations, as the present study focused on practicing dentists, whereas Badrasawi et al. investigated patients. The present study also found no statistically significant association between the type of university from which dentists graduated and their mean knowledge and behavior scores related to nutrition and oral health. This finding contrasts with the results reported by Seyed Moallemi, who identified a significant relationship between nutritional awareness and university affiliation [14]. This discrepancy may be attributed to differences in study populations, as Seyed Moallemi evaluated students from both medical and non-medical universities, whereas all participants in the present study were graduates of medical sciences universities, with variation limited to institutional type rather than educational discipline. Finally, consistent with the findings of Seyed Moallemi, no significant association was observed between behavior related to nutrition and oral health and the type of university attended. Moreover, the results indicated no statistically significant relationship between dentists’ knowledge and behavior regarding nutrition and oral health and their professional practice setting in Tehran at the pre-intervention stage.

Despite the promising findings, several limitations of the present study can be pointed out. The sample was drawn from general dentists in Tehran using convenience sampling, which may limit generalizability. Dentists in other regions or with different professional backgrounds might exhibit different educational needs or responses to intervention. Moreover, participants were recruited through professional social-media platforms, which may have introduced selection bias toward dentists who are more digitally active and engaged, and further limits generalizability to practitioners with lower social-media use. In addition, behavioral outcomes were based entirely on self-report rather than observed clinical practice, which may be subject to social desirability bias and may not accurately reflect actual counseling behavior in clinical settings; future studies should consider incorporating objective or observed measures of counseling behavior. A further limitation is that allocation concealment was not implemented, since randomization and intervention delivery were performed by the same investigator; future studies should consider assigning these roles to separate, blinded personnel to minimize potential selection bias. Another limitation is the relatively short duration of the intervention and follow-up. Future research should incorporate longitudinal follow-up to determine whether improvements persist over time. The two groups also differed significantly at baseline in dental school type and workplace setting; although covariate-adjusted analysis found no significant association between these factors and pre-intervention knowledge or practice scores, residual confounding cannot be fully excluded.

Overall, this study demonstrates that digital educational interventions can significantly enhance dentists’ knowledge and behavior regarding nutrition and oral health. The findings support integrating multimedia-based learning into continuing dental education programs and highlight the need for ongoing professional development in this critical area. As oral diseases remain highly prevalent worldwide, equipping dental practitioners with evidence-based nutritional knowledge contributes substantially to preventive care and public health. The enhanced effectiveness of multimedia learning suggests that educational institutions and professional organizations should consider incorporating multimodal instructional strategies to maximize learning outcomes.

## Conclusion

This study demonstrates that digital educational interventions improve general dentists’ knowledge and self-reported behavior regarding nutrition and oral health, with multimedia-based education showing greater effectiveness than audio-only formats. The superiority of multimedia suggests that integrating visual and auditory elements enhances learning efficiency, while the lack of association between demographic variables and outcomes indicates that such interventions are beneficial across diverse practitioner profiles and supports the inclusion of multimedia nutrition education in continuing dental education programs to strengthen preventive oral health care.
