# Supplementary material for: Multimedia versus audio-based education for improving dentists’ nutrition-related knowledge and behavior: A randomized comparative study
Source: PLoS One. 2026 Oct 28;21(10):e0360426. doi: 10.1371/journal.pone.0360426 (PMC13716169; doi:10.1371/journal.pone.0360426)
Supplement: Supplementary file 1 [file pone.0360426.s001.docx]

Major comments

1. Randomization procedure needs more detail

- The manuscript states that participants were "randomly assigned using a simple random allocation procedure" but does not explain:
- How randomization was generated (computer-generated sequence, random number table, etc.)
- Allocation concealment
- Who generated the sequence and assigned participants
- Please provide details consistent with CONSORT reporting standards.

2. Convenience sampling limits external validity

Participants were recruited through social media platforms using convenience sampling. This introduces selection bias and limits generalizability beyond digitally active dentists in Tehran. This limitation should be explicitly acknowledged.

3. Sample size calculation is inadequate

The sample size section is insufficient. The manuscript merely states that 166 dentists were adequate for detecting moderate effect sizes.

Authors should provide:

- Expected effect size
- Alpha level
- Statistical power
- Formula or software used (e.g., G*Power)
- Anticipated attrition rate

4. Questionnaire validation requires more detail

Although face and content validity are described, psychometric properties remain incomplete.

Please report:

- Actual CVI and CVR values
- Reliability statistics (Cronbach’s alpha)
- Test–retest reliability
- Factor analysis, if performed

Without reliability measures, confidence in the instrument is limited.

5. Educational intervention description is insufficient

The intervention is described only briefly.

Please provide:

- Exact content/modules covered
- Number of videos/audio files
- Duration of each session
- Whether participants completed all modules
- Measures of adherence or engagement analytics

A five-minute intervention appears rather short to produce substantial behavioral changes.

6. Behavioral outcomes are self-reported

The study evaluates self-reported behaviors rather than actual clinical practices. This should be emphasized as a limitation due to possible social desirability bias.

7. Statistical analysis needs strengthening

The abstract reports repeated-measures ANOVA, while the methods describe paired t-tests and ANCOVA. This inconsistency must be resolved.

Additionally, authors should report:

- Effect sizes (Cohen’s d or partial eta squared)
- Confidence intervals
- Assumptions of normality
- Handling of missing data

8. Baseline comparability between groups

- Table 1 presents demographics but does not provide p-values comparing groups.
- Please include statistical tests demonstrating baseline equivalence.

9. CONSORT flow diagram missing

- As this is an RCT, a CONSORT flowchart should be included showing:
- Assessed for eligibility
- Randomized
- Lost to follow-up
- Analyzed

10. Discussion should be expanded

- The discussion should compare findings with prior studies on:
- Digital learning in dentistry
- Multimedia learning theory
- Continuing dental education interventions

Mechanisms explaining why multimedia outperformed audio should also be discussed.

**Minor Comments**

Language and style

1.Title:

Consider changing:

“nutrition and oral Health”

to

“nutrition and oral health”

2,Page 6:

There is an unintended punctuation mark:

"obtained.، The mean necessity score..."

Remove the Arabic comma.

3.Page 7:

"the role of length"

This appears erroneous and should likely be revised.

4.References:

Ensure all references follow PLOS ONE style.

5.Tables:

Add statistical comparisons and effect sizes where appropriate.

**Strengths of the Study**

Relevant and novel topic.

Randomized controlled design.

Open data availability through Zenodo.

Practical implications for continuing dental education.

Focus on evidence-based nutrition counseling.
