# Supplementary material for: Multimedia versus audio-based education for improving dentists’ nutrition-related knowledge and behavior: A randomized comparative study
Source: PLoS One. 2026 Oct 28;21(10):e0360426. doi: 10.1371/journal.pone.0360426 (PMC13716169; doi:10.1371/journal.pone.0360426)
Supplement: Supplementary file 2 [file pone.0360426.s003.docx]

The manuscript has been thoroughly reviewed, and all necessary changes which were recommended, have been made in accordance with the reviewers’ comments. Revisions have been incorporated to improve clarity, accuracy, and overall quality. The updated version now reflects the required modifications.
