# Supplementary material for: Multimedia versus audio-based education for improving dentists’ nutrition-related knowledge and behavior: A randomized comparative study
Source: PLoS One. 2026 Oct 28;21(10):e0360426. doi: 10.1371/journal.pone.0360426 (PMC13716169; doi:10.1371/journal.pone.0360426)
Supplement: Peer review history

# Peer review: Decision Letter 0

**DOI:** 10.1371/journal.pone.0360426.r001

**Authors:** Tanay Chaubal

21 Jun 2026

Dear Dr. Gholami,

Thank you for submitting your manuscript to PLOS ONE. After careful consideration, we feel that it has merit but does not fully meet PLOS ONE’s publication criteria as it currently stands. Therefore, we invite you to submit a revised version of the manuscript that addresses the points raised during the review process.

==============================

**ACADEMIC EDITOR:** **I thank you and your efforts in writing the manuscript. However, there are queries raised by the reviewers' which need to be addressed to make your manuscript more robust.**

==============================

Please submit your revised manuscript by Aug 20 2026 11:59PM. If you will need more time than this to complete your revisions, please reply to this message or contact the journal office at plosone@plos.org. When you're ready to submit your revision, log on to https://www.editorialmanager.com/pone/ and select the 'Submissions Needing Revision' folder to locate your manuscript file.

- A letter that responds to each point raised by the academic editor and reviewer(s). You should upload this letter as a separate file labeled 'Response to Reviewers'.
- A marked-up copy of your manuscript that highlights changes made to the original version. You should upload this as a separate file labeled 'Revised Manuscript with Track Changes'.
- An unmarked version of your revised paper without tracked changes. You should upload this as a separate file labeled 'Manuscript'.

If you would like to make changes to your financial disclosure, please include your updated statement in your cover letter. Guidelines for resubmitting your figure files are available below the reviewer comments at the end of this letter.

If applicable, we recommend that you deposit your laboratory protocols in protocols.io to enhance the reproducibility of your results. Protocols.io assigns your protocol its own identifier (DOI) so that it can be cited independently in the future. For instructions see: https://journals.plos.org/plosone/s/submission-guidelines#loc-laboratory-protocols. Additionally, PLOS ONE offers an option for publishing peer-reviewed Lab Protocol articles, which describe protocols hosted on protocols.io. Read more information on sharing protocols at https://plos.org/protocols?utm_medium=editorial-email&utm_source=authorletters&utm_campaign=protocols.

As the corresponding author, your ORCID iD is verified in the submission system and will appear in the published article. PLOS supports the use of ORCID, and we encourage all coauthors to register for an ORCID iD and use it as well. Please encourage your coauthors to verify their ORCID iD within the submission system before final acceptance, as unverified ORCID iDs will not appear in the published article. *Only*  the individual author can complete the verification step; PLOS staff *cannot*  verify ORCID iDs on behalf of authors.

We look forward to receiving your revised manuscript.

Kind regards,

Tanay Chaubal

Academic Editor

PLOS One

Journal Requirements:

When submitting your revision, we need you to address these additional requirements.

1. Please ensure that your manuscript meets PLOS ONE's style requirements, including those for file naming. The PLOS ONE style templates can be found at

https://journals.plos.org/plosone/s/file?id=wjVg/PLOSOne_formatting_sample_main_body.pdf and https://journals.plos.org/plosone/s/file?id=ba62/PLOSOne_formatting_sample_title_authors_affiliations.pdf

2. In your Methods section, please include additional information about your dataset and ensure that you have included a statement specifying whether the collection and analysis method complied with the terms and conditions for the source of the data.

3. Your ethics statement should only appear in the Methods section of your manuscript. If your ethics statement is written in any section besides the Methods, please delete it from any other section.

4. We note you have included a table to which you do not refer in the text of your manuscript. Please ensure that you refer to Table 1 in your text; if accepted, production will need this reference to link the reader to the Table.

5. If the reviewer comments include a recommendation to cite specific previously published works, please review and evaluate these publications to determine whether they are relevant and should be cited. There is no requirement to cite these works unless the editor has indicated otherwise.

[Note: HTML markup is below. Please do not edit.]

Reviewers' comments:

Reviewer's Responses to Questions

**Comments to the Author**

1. Is the manuscript technically sound, and do the data support the conclusions?

Reviewer #1: Partly

Reviewer #2: Yes

Reviewer #3: Yes

**********

2. Has the statistical analysis been performed appropriately and rigorously?

Reviewer #1: No

Reviewer #2: Yes

Reviewer #3: No

**********

3. Have the authors made all data underlying the findings in their manuscript fully available?

The PLOS Data policy

Reviewer #1: Yes

Reviewer #2: Yes

Reviewer #3: Yes

**********

4. Is the manuscript presented in an intelligible fashion and written in standard English?

Reviewer #1: Yes

Reviewer #2: Yes

Reviewer #3: Yes

**********

Reviewer #1: Overall Evaluation

The manuscript addresses an important and relevant topic in dental education and preventive oral health. Comparing multimedia and audio-based educational interventions among practicing dentists is timely and valuable initiative. But there are concerns related to language, organization, methodological clarity, statistical reporting, academic writing style, and formatting that need substantial improvement.

1. Manuscript Formatting and Structure

The manuscript appears to contain inconsistent formatting, including bullet-style writing and uneven paragraph organization.

The Introduction and Discussion require restructuring into coherent thematic paragraphs.

Recommendation:

Convert all bullet-like sections into formal academic prose.

Ensure each paragraph addresses one central idea.

Follow standard IMRAD structure consistently.

2. Title Improvement

3. Introduction Needs Stronger Focus

Introduction is overly descriptive. Multiple paragraphs contain excessive references (e.g., 7 references in a single paragraph). Some information is repetitive. Research gap is not sharply defined. Add a stronger concluding rationale and hypothesis statement. Update with more recent evidence (2024–2026 if available). Standardize all references according to Vancouver style.

4. Methodology Requires Clarification

Important Missing Details:

Randomization procedure is insufficiently described.

No CONSORT flow diagram.

Sample size calculation lacks formula and assumptions.

Reliability testing is missing.

Pilot testing details

6. Statistical Analysis Concerns

Repeated-measures ANOVA was mentioned in the abstract but not clearly described in Methods.

7. Results Section Needs Refinement

Results are overly descriptive.

Tables are large and difficult to interpret.

Some findings are repeated in text and tables.

8. Discussion is too descriptive and repetitive.

Limited theoretical interpretation.

Decision: Major Revision

The manuscript addresses an important topic and has publication potential; however, substantial revisions are required in:

academic writing,

methodological reporting,

statistical clarity,

discussion depth,

reference quality,

formatting consistency.

Reviewer #2: This randomized controlled educational study evaluates an important topic in preventive dentistry and continuing professional education. The comparison of multimedia and audio-based learning approaches is timely and relevant, and the findings demonstrate that both interventions improved dentists nutrition-related knowledge and self-reported behaviors, with greater improvements observed in the multimedia group.

Please provide additional details regarding the randomization process ( method used for sequence generation and allocation concealment).

Clarify whether participant engagement with the educational materials was objectively monitored or based on self-report.

Report effect sizes alongside p-values to better demonstrate the magnitude of the intervention effect.

A brief discussion of the limitations associated with self-reported behavioral outcomes would strengthen the manuscript.

Minor English language editing is recommended to improve readability and correct a few grammatical inconsistencies.

Reviewer #3: Major comments

1. Randomization procedure needs more detail

• The manuscript states that participants were "randomly assigned using a simple random allocation procedure" but does not explain:

• How randomization was generated (computer-generated sequence, random number table, etc.)

• Allocation concealment

• Who generated the sequence and assigned participants

• Please provide details consistent with CONSORT reporting standards.

2. Convenience sampling limits external validity

Participants were recruited through social media platforms using convenience sampling. This introduces selection bias and limits generalizability beyond digitally active dentists in Tehran. This limitation should be explicitly acknowledged.

3. Sample size calculation is inadequate

The sample size section is insufficient. The manuscript merely states that 166 dentists were adequate for detecting moderate effect sizes.

Authors should provide:

• Expected effect size

• Alpha level

• Statistical power

• Formula or software used (e.g., G*Power)

• Anticipated attrition rate

4. Questionnaire validation requires more detail

Although face and content validity are described, psychometric properties remain incomplete.

Please report:

• Actual CVI and CVR values

• Reliability statistics (Cronbach’s alpha)

• Test–retest reliability

• Factor analysis, if performed

Without reliability measures, confidence in the instrument is limited.

5. Educational intervention description is insufficient

The intervention is described only briefly.

Please provide:

• Exact content/modules covered

• Number of videos/audio files

• Duration of each session

• Whether participants completed all modules

• Measures of adherence or engagement analytics

A five-minute intervention appears rather short to produce substantial behavioral changes.

6. Behavioral outcomes are self-reported

The study evaluates self-reported behaviors rather than actual clinical practices. This should be emphasized as a limitation due to possible social desirability bias.

7. Statistical analysis needs strengthening

The abstract reports repeated-measures ANOVA, while the methods describe paired t-tests and ANCOVA. This inconsistency must be resolved.

Additionally, authors should report:

• Effect sizes (Cohen’s d or partial eta squared)

• Confidence intervals

• Assumptions of normality

• Handling of missing data

8. Baseline comparability between groups

• Table 1 presents demographics but does not provide p-values comparing groups.

• Please include statistical tests demonstrating baseline equivalence.

9. CONSORT flow diagram missing

• As this is an RCT, a CONSORT flowchart should be included showing:

• Assessed for eligibility

• Randomized

• Lost to follow-up

• Analyzed

10. Discussion should be expanded

• The discussion should compare findings with prior studies on:

• Digital learning in dentistry

• Multimedia learning theory

• Continuing dental education interventions

Mechanisms explaining why multimedia outperformed audio should also be discussed.

Minor Comments

Language and style

1.Title:

Consider changing:

“nutrition and oral Health”

to

“nutrition and oral health”

2,Page 6:

There is an unintended punctuation mark:

"obtained.، The mean necessity score..."

Remove the Arabic comma.

3.Page 7:

"the role of length"

This appears erroneous and should likely be revised.

4.References:

Ensure all references follow PLOS ONE style.

5.Tables:

Add statistical comparisons and effect sizes where appropriate.

Strengths of the Study

Relevant and novel topic.

Randomized controlled design.

Open data availability through Zenodo.

Practical implications for continuing dental education.

Focus on evidence-based nutrition counseling.

**********

what does this mean?). If published, this will include your full peer review and any attached files.

If you choose “no”, your identity will remain anonymous but your review may still be made public.

**Do you want your identity to be public for this peer review?**  For information about this choice, including consent withdrawal, please see our Privacy Policy

Reviewer #1: **Yes:**  Dr. Sarah Ali

Reviewer #2: **Yes:**  Akshay Ashok Katara

Reviewer #3: **Yes:**  Dr Sarah Paul

**********

[NOTE: If reviewer comments were submitted as an attachment file, they will be attached to this email and accessible via the submission site. Please log into your account, locate the manuscript record, and check for the action link "View Attachments". If this link does not appear, there are no attachment files.]

To ensure your figures meet our technical requirements, please review our figure guidelines: https://journals.plos.org/plosone/s/figures

You may also use PLOS’s free figure tool, NAAS, to help you prepare publication quality figures: https://journals.plos.org/plosone/s/figures#loc-tools-for-figure-preparation.

NAAS will assess whether your figures meet our technical requirements by comparing each figure against our figure specifications.

Attachment Submitted filename: Major comments.docx

# Author comment: Author response to Decision Letter 1

**DOI:** 10.1371/journal.pone.0360426.r002

18 Aug 2026

Response to Reviewers

Manuscript: "Effectiveness of Multimedia versus Audio-Based Educational Interventions on Dentists' Knowledge and Behavior Regarding Nutrition and Oral Health"

We thank the editor and all three reviewers for their careful reading of the manuscript and their constructive comments, which have substantially improved its clarity, methodological transparency, and academic writing style. Below we address each comment individually and in full. All changes have been recorded as Word tracked changes in the revised manuscript file.

■ DONE = fully completed and verified.

Editor's comments

Comment: Please ensure that your manuscript meets PLOS ONE's style requirements, including those for file naming.

Response — DONE: The manuscript has been checked against the PLOS ONE formatting templates (main body and title/authors/affiliations).

Comment: In your Methods section, please include additional information about your dataset and ensure that you have included a statement specifying whether the collection and analysis method complied with the terms and conditions for the source of the data.

Response — DONE: The desired details were added to the method (Page 6, Line 121-123).

Comment: Your ethics statement should only appear in the Methods section of your manuscript. If your ethics statement is written in any section besides the Methods, please delete it from any other section.

Response — DONE: We checked the full manuscript: the ethics statement appears in exactly two places, both inside the Methods section (the Study Design paragraph and the dedicated "Ethical Considerations" subsection). No ethics-related text appears in the Abstract, Introduction, Results, Discussion, or Conclusion.

Comment: We note you have included a table to which you do not refer in the text of your manuscript. Please ensure that you refer to Table 1 in your text; if accepted, production will need this reference to link the reader to the Table.

Response — DONE: A sentence — "Demographic characteristics of participants are presented in Table 1." — has been inserted at the end of the Participant Characteristics paragraph in Results.

→ Results → Page11, Line 233

Comment: If the reviewer comments include a recommendation to cite specific previously published works, please review and evaluate these publications to determine whether they are relevant and should be cited. There is no requirement to cite these works unless the editor has indicated otherwise.

Response — DONE: None of the three reviewers recommended citation of specific external.

Reviewer #1

Overall Evaluation: The manuscript addresses an important and relevant topic; concerns relate to language, organization, methodological clarity, statistical reporting, academic writing style, and formatting.

Response — DONE: We appreciate the reviewer's positive overall assessment. We have revised the manuscript for academic writing style, methodological clarity, and statistical reporting, detailed point-by-point below.

1. Manuscript Formatting and Structure

Comment: The manuscript appears to contain inconsistent formatting, including bullet-style writing and uneven paragraph organization. The Introduction and Discussion require restructuring into coherent thematic paragraphs. Convert all bullet-like sections into formal academic prose; ensure each paragraph addresses one central idea; follow standard IMRAD structure consistently.

Response — DONE: We have restructured the Introduction and Discussion into distinct thematic paragraphs following a standard IMRaD progression. All bullet-like sections have been converted into formal academic prose in the Methods. In the Discussion section, a new, more detailed paragraph was added that interpreted the results through the cognitive theory of multimedia learning—giving the discussion a clearer thematic separation between (1) the purpose and main findings, (2) the theoretical interpretation of the findings and comparison with the previous literature, (3) the results regarding the independent variables and comparison with the previous literature, (4) limitations, and (5) suggestions. The limitations paragraph was also expanded with additional points and clearer details.

→ Methods (bullet conversion); Page 8 –Discussion, Page 17, Line 315, new paragraph (theory/mechanism); Page 19-20 – limitations paragraph (expanded)

2. Title Improvement

Comment: Title Improvement.

Response — DONE: The title has been revised to more clearly reflect the study design and outcomes, as follows:

“Multimedia versus audio-based education for improving dentists' nutrition-related knowledge and behavior: A randomized comparative study”

3. Introduction Needs Stronger Focus

Comment: Introduction is overly descriptive. Multiple paragraphs contain excessive references (e.g., 7 references in a single paragraph). Some information is repetitive. Research gap is not sharply defined. Add a stronger concluding rationale and hypothesis statement. Update with more recent evidence (2024–2026 if available). Standardize all references according to Vancouver style.

Response — DONE: Two recent references (2024) were added to both the introduction and discussion - Ijaz et al. 2024 (a randomized trial of digitally presented case-based learning in dental students) and Mayer 2024 (an updated account of the cognitive theory of multimedia learning). We also re-checked each citation in the manuscript for the "excessive references in a paragraph" issue: the extra references were removed (Line 58). The research gap has been more sharply defined and the introduction now closes with an explicit a priori hypothesis (Page 4 – Introduction, final paragraph).

4. Methodology Requires Clarification

Comment: Important Missing Details: Randomization procedure is insufficiently described. No CONSORT flow diagram. Sample size calculation lacks formula and assumptions. Reliability testing is missing. Pilot testing details.

Response — DONE: (a) We have expanded the methods section to describe the randomization procedure in detail (Page 6, Line 112). We have also acknowledged the absence of allocation concealment as a limitation of the study in the discussion section (Page 19, Line 368). (b) A CONSORT flow diagram (Fig 1) has been inserted directly after the Study Design paragraph (Page 5). (c) More information about the sample size has been added (Page 8, Line 178). (d) We have explained more about the pilot study and supplied the reliability data (Study Instruments, Line 144).

5. Statistical Analysis Concerns

Comment: Repeated-measures ANOVA was mentioned in the abstract but not clearly described in Methods.

Response — DONE: Corrections related to analysis methods have been made in the Methods and Summary.

→ Page 10 – Methods, Statistical Analysis

6. Results Section Needs Refinement

Comment: Results are overly descriptive. Tables are large and difficult to interpret. Some findings are repeated in text and tables.

Response — DONE: Tables 1, 2, and 3 have been made more compact in terms of design and layout to make them easier to read for the audience. A p-value column (chi-square tests, computed from the group counts in the underlying study data) has been added to Table 1. In order to make the tables more analytical, Table 4 regarding the paired t-test results (previously only its explanation was written in the text) was added. Explanations regarding ANOVA analysis were also added at the end of the results text.

We also corrected an internal inconsistency in the Results, where the ANCOVA sentence referred to "pre-intervention scores" when it should read "post-intervention scores"; this is now fixed.

→ Table 4; Page 16 – ANOVA Results; Page 16, Line 287

7. Discussion is too descriptive and repetitive; limited theoretical interpretation

Comment: Discussion is too descriptive and repetitive. Limited theoretical interpretation.

Response — DONE: Discussion has been restructured. a new paragraph was added providing theoretical interpretation of the multimedia advantage via the cognitive theory of multimedia learning, citing Mayer (2024) and Ijaz et al. (2024) → Page 17. The limitations paragraph was also expanded with additional points and clearer details → Page 19.

Reviewer #2

Comment: Please provide additional details regarding the randomization process (method used for sequence generation and allocation concealment).

Response — DONE: We have expanded the methods section to describe the randomization procedure in detail (Page 6, Line 112). We have also acknowledged the absence of allocation concealment as a limitation of the study in the discussion section (Page 19, Line 368).

Comment: Clarify whether participant engagement with the educational materials was objectively monitored or based on self-report.

Response — DONE: Two to three reminder text messages were sent to participants during the one-month intervention period, but no read-receipts, view analytics, or completion tracking were used. This has been added explicitly in the Data Collection subsection of Methods (Page 9, Line 200), and is now also flagged as a limitation in the Discussion. (Discussion; Page 19, Line 362)

Comment: Report effect sizes alongside p-values to better demonstrate the magnitude of the intervention effect.

Response — DONE: Effect sizes (Cohen's d) have been added to Table 4 for all within-group paired comparisons (range: 1.20–1.55) and reported in the text for the between-group comparisons (d = 0.96 for knowledge, d = 0.45 for behavior). Partial eta squared has also been added for the ANOVA results (time: η²p = 0.688; group: η²p = 0.027; time × intervention: η²p = 0.171). These changes are reflected in the revised Results section (page 15, 16) and Table 4.

Comment: A brief discussion of the limitations associated with self-reported behavioral outcomes would strengthen the manuscript.

Response — DONE: A sentence has been inserted into the Discussion limitations paragraph: In addition, behavioral outcomes were based entirely on self-report rather than observed clinical practice, which may be subject to social desirability bias and may not accurately reflect actual counseling behavior in clinical settings; future studies should consider incorporating objective or observed measures of counseling behavior

→ Discussion; Page 19, Line 365.

Comment: Minor English language editing is recommended to improve readability and correct a few grammatical inconsistencies.

Response — DONE: English language editing was done.

Reviewer #3

Major Comments

1. Randomization procedure needs more detail

Comment: The manuscript states that participants were "randomly assigned using a simple random allocation procedure" but does not explain how randomization was generated, allocation concealment, or who generated the sequence and assigned participants. Please provide details consistent with CONSORT reporting standards.

Response — DONE: We have expanded the methods section to describe the randomization procedure in detail (Page 6, Line 112). We have also acknowledged the absence of allocation concealment as a limitation of the study in the discussion section (Page 19, Line 368).

2. Convenience sampling limits external validity

Comment: Participants were recruited through social media platforms using convenience sampling. This introduces selection bias and limits generalizability beyond digitally active dentists in Tehran. This limitation should be explicitly acknowledged.

Response — DONE: A new sentence has been inserted directly into the limitations paragraph, explicitly naming social-media recruitment as a source of selection bias toward dentists who are more digitally active and engaged, and noting that this further limits generalizability to practitioners with lower social-media use.

→ Page 19, Line 363 – Discussion, limitations paragraph

3. Sample size calculation is inadequate

Comment: The sample size section is insufficient. Authors should provide expected effect size, alpha level, statistical power, formula or software used (e.g., G*Power), and anticipated attrition rate.

Response — DONE: The Sample Size paragraph now has been amended: Sample size was calculated using PASS version 11 software (Repeated Measures ANOVA Power Analysis option), based on the effect-size assumptions reported in a prior study on nutrition-related oral health knowledge and practice [14], with α = 0.05. A sample size of 83 dentists per group provided 80.3% power to detect the between-group (intervention type) effect (Sigma = 0.87) and 99.9% power to detect the within-group, pre-post effect (Sigma = 0.5).

→ Page 8, Sample Size, Line 178

4. Questionnaire validation requires more detail

Comment: Although face and content validity are described, psychometric properties remain incomplete. Please report actual CVI and CVR values, reliability statistics (Cronbach's alpha), test-retest reliability, and factor analysis if performed.

Response — DONE: The requested validity (CVI, CVR) and reliability (Cronbach's alpha, test-retest ICC) values have been added to the Methods section.

→ Page 7, Line 137– Study Instruments

5. Educational intervention description is insufficient

Comment: Please provide exact content/modules covered, number of videos/audio files, duration of each session, whether participants completed all modules, and measures of adherence or engagement analytics. A five-minute intervention appears rather short to produce substantial behavioral changes.

Response — DONE: “Data Collection” section now specifies: one narrated video (multimedia arm) and one audio file (audio arm), each approximately 5 minutes, covering the same content — the relationship between healthy nutrition and periodontal health, the role of sugar consumption in caries formation, prevention of dental caries, oral cancer, and tooth wear, and the effects of cariostatic and anticariogenic foods — delivered over one month via WhatsApp, Telegram, and Bale, with 2-3 reminder messages sent during that period. Engagement was assessed based on participants' self-report, supported by the reminder messages; no objective tracking (e.g., read-receipts, view analytics, or completion tracking) was used. This has been added explicitly in the Data Collection subsection of Methods (Page 9), and the absence of objective engagement tracking is also flagged as a limitation in the Discussion) Page 19, Line 368(. The short duration of intervention and follow-up was already mentioned in the limitations section of the discussion (Line 371). →

6. Behavioral outcomes are self-reported

Comment: The study evaluates self-reported behaviors rather than actual clinical practices. This should be emphasized as a limitation due to possible social desirability bias.

Response — DONE: A sentence naming self-report and social desirability bias as a limitation has been inserted into the Discussion limitations paragraph.

→ Page 19, Line 366 – Discussion, limitations paragraph

7. Statistical analysis needs strengthening

Comment: The abstract reports repeated-measures ANOVA, while the methods describe paired t-tests and ANCOVA. This inconsistency must be resolved. Additionally, authors should report effect sizes, confidence intervals, assumptions of normality, and handling of missing data.

Response — DONE: Inconsistency between abstract and methods: We have revised the abstract to accurately reflect the statistical methods described in the Methods section. The abstract now reports paired t-tests, repeated-measures ANOVA, and ANCOVA consistently with the main text.

Effect sizes (Cohen's d) have been added to Table 4 for all within-group paired comparisons (range: 1.20–1.55) and reported in the text for the between-group comparisons (d = 0.96 for knowledge, d = 0.45 for behavior). Partial eta squared has also been added for the ANOVA results (time: η²p = 0.688; group: η²p = 0.027; time × intervention: η²p = 0.171). These changes are reflected in the revised Results section (page 15, 16) and Table 4. In addition, 95% confidence intervals for the mean change in each paired t-test have been added to Table 4.

Missing data: No missing data were present in this study, as convenience sampling was continued until the pre-determined sample size was achieved; and all participants who completed the baseline questionnaire also completed the post-intervention questionnaire; therefore, no missing data handling procedures were required. This has been clarified in the Methods section (Page 10, Line 201).

8. Baseline comparability between groups

Comment: Table 1 presents demographics but does not provide p-values comparing groups. Please include statistical tests demonstrating baseline equivalence.

Response — DONE: A p-value column has been added to Table 1 and related information has been added to result part (Page 12, Line 238): As shown in Table 1, the audio-based and multimedia groups did not differ significantly in gender (p=0.534) or years since graduation (p=0.432), but did differ significantly in dental school type (p<0.001) and workplace (p=0.001). The two significant differences (dental school type, workplace) are now explicitly noted as a limitation in the Discussion, alongside a note that covariate-adjusted analysis found no significant association between these factors and pre-intervention scores.

→ Page 20, Line 373 – Discussion, limitations

9. CONSORT flow diagram missing

Comment: As this is an RCT, a CONSORT flowchart should be included showing assessed for eligibility, randomized, lost to follow-up, and analyzed.

Response — DONE: A CONSORT flow diagram (Fig 1) has been inserted as a new figure immediately after the Study Design paragraph. (Page 5 – Study Design)

10. Discussion should be expanded

Comment: The discussion should compare findings with prior studies on digital learning in dentistry, multimedia learning theory, and continuing dental education interventions. Mechanisms explaining why multimedia outperformed audio should also be discussed.

Response — DONE: A new paragraph has been inserted explaining the multimedia advantage through the cognitive theory of multimedia learning, citing Mayer (2024) and Ijaz et al. (2024), a comparable 2024 randomized trial of digital case-based learning in dental students.

→ Page 17 – Discussion

Minor Comments

Title

Comment: Consider changing "nutrition and oral Health" to "nutrition and oral health".

Response — DONE: The title has been improved and revised to more clearly reflect the study design and outcomes, as follows:

Multimedia versus audio-based education for improving dentists' nutrition-related knowledge and behavior: A randomized comparative study

Page 6

Comment: There is an unintended punctuation mark: "obtained، the mean necessity score..." Remove the Arabic comma.

Response — DONE: To provide more complete information, the entire paragraph has been revised. Addressing the point raised by the reviewer as well.

Page 7

Comment: "the role of length" — This appears erroneous and should likely be revised.

Response — DONE: The intervention content list corresponds to "the relationship between healthy nutrition and periodontal health, the role of sugar consumption in caries formation, prevention of dental caries, oral cancer, and tooth wear, and the effects of cariostatic and anticariogenic foods." The phrase has accordingly been corrected to "the role of sugar consumption in caries formation."

References

Comment: Ensure all references follow PLOS ONE style.

Response — DONE: We checked every reference in the list against PLOS ONE's Vancouver-based format and amended the incomplete references.

Tables

Comment: Add statistical comparisons and effect sizes where appropriate.

Response — DONE: Table 4 regarding the paired t-test results (previously only its explanation was written in the text) was added. Explanations regarding ANOVA analysis were also added at the end of the results text. Effect sizes (Cohen's d) have been added to Table 4 for all within-group paired comparisons (range: 1.20–1.55) and reported in the text for the between-group comparisons (d = 0.96 for knowledge, d = 0.45 for behavior). Partial eta squared has also been added for the ANOVA results (time: η²p = 0.688; group: η²p = 0.027; time × intervention: η²p = 0.171). These changes are reflected in the revised Results section (page 15, 16) and Table 4.

Reference numbers have changed throughout the text.

Attachment Submitted filename: Response_to_Reviewers.docx

# Peer review: Decision Letter 1

**DOI:** 10.1371/journal.pone.0360426.r003

**Authors:** Tanay Chaubal

27 Sep 2026

Multimedia versus audio-based education for improving dentists' nutrition-related knowledge and behavior: A randomized comparative study

PONE-D-26-21959R1

Dear Dr. Mahdia Gholami,

We’re pleased to inform you that your manuscript has been judged scientifically suitable for publication and will be formally accepted for publication once it meets all outstanding technical requirements.

Within one week, you’ll receive an e-mail detailing the required amendments. When these have been addressed, you’ll receive a formal acceptance letter and your manuscript will be scheduled for publication.

An invoice will be generated when your article is formally accepted. Please note, if your institution has a publishing partnership with PLOS and your article meets the relevant criteria, all or part of your publication costs will be covered. Please make sure your user information is up-to-date by logging into Editorial Manager at Editorial Manager® and clicking the ‘Update My Information' link at the top of the page. For questions related to billing, please contact billing support.

If your institution or institutions have a press office, please notify them about your upcoming paper to help maximize its impact. If they’ll be preparing press materials, please inform our press team as soon as possible -- no later than 48 hours after receiving the formal acceptance. Your manuscript will remain under strict press embargo until 2 pm Eastern Time on the date of publication. For more information, please contact onepress@plos.org.

Kind regards,

Tanay Chaubal

Academic Editor

PLOS One

Additional Editor Comments (optional):

Reviewers' comments:

Reviewer's Responses to Questions

**Comments to the Author**

Reviewer #1: All comments have been addressed

Reviewer #2: All comments have been addressed

**********

2. Is the manuscript technically sound, and do the data support the conclusions?

Reviewer #1: Yes

Reviewer #2: Yes

**********

3. Has the statistical analysis been performed appropriately and rigorously?

Reviewer #1: Yes

Reviewer #2: Yes

**********

4. Have the authors made all data underlying the findings in their manuscript fully available?

The PLOS Data policy

Reviewer #1: Yes

Reviewer #2: Yes

**********

5. Is the manuscript presented in an intelligible fashion and written in standard English?

Reviewer #1: Yes

Reviewer #2: Yes

**********

Reviewer #1: Recommendation:

I have carefully reviewed the article in detail and assessed the manuscript in light of the comments and recommendations provided. The necessary revisions have been incorporated throughout the manuscript to improve its clarity, coherence, scientific accuracy, and overall quality. Relevant sections have been revised, and appropriate corrections and additions have been made wherever required. The methodology, presentation of results, discussion, and conclusion have also been reviewed to ensure consistency and alignment with the objectives of the study. Additionally, grammatical, typographical, and formatting errors have been corrected, and the references have been checked and updated where necessary. I believe that the revised manuscript addresses the major concerns and suggestions raised during the review process and is now substantially improved. I have made every effort to ensure that the revised article meets the required academic and scientific standards and is suitable for further consideration.

Reviewer #2: I thank the authors for the opportunity to examine the revised manuscript. They have put in a most commendable amount of work in responding to the reviewers comments, and the manuscript has been greatly improved with regard to clarity, organization, and the general presentation. The revisions have enhanced the methodology, improved the discussion, and led to a better interpretation of the findings. The manuscript is well written, the results are clearly set out, and the conclusions are supported by the data.

**********

what does this mean?). If published, this will include your full peer review and any attached files.

If you choose “no”, your identity will remain anonymous but your review may still be made public.

**Do you want your identity to be public for this peer review?**  For information about this choice, including consent withdrawal, please see our Privacy Policy

Reviewer #1: **Yes:**  Dr. Sarah Ali

Reviewer #2: **Yes:**  Akshay Katara

**********

Attachment Submitted filename: Reviewer comments (2).docx

# Editor report: Acceptance letter

**DOI:** 10.1371/journal.pone.0360426.r004

**Authors:** Tanay Chaubal

PONE-D-26-21959R1

PLOS One

Dear Dr. Gholami,

I'm pleased to inform you that your manuscript has been deemed suitable for publication in PLOS One. Congratulations! Your manuscript is now being handed over to our production team.

At this stage, our production department will prepare your paper for publication. This includes ensuring the following:

* All references, tables, and figures are properly cited

* All relevant supporting information is included in the manuscript submission,

* There are no issues that prevent the paper from being properly typeset

You will receive further instructions from the production team, including instructions on how to review your proof when it is ready. Please keep in mind that we are working through a large volume of accepted articles, so please give us a few days to review your paper and let you know the next and final steps.

Lastly, if your institution or institutions have a press office, please let them know about your upcoming paper now to help maximize its impact. If they'll be preparing press materials, please inform our press team within the next 48 hours. Your manuscript will remain under strict press embargo until 2 pm Eastern Time on the date of publication. For more information, please contact onepress@plos.org.

You will receive an invoice from PLOS for your publication fee after your manuscript has reached the completed accept phase. If you receive an email requesting payment before acceptance or for any other service, this may be a phishing scheme. Learn how to identify phishing emails and protect your accounts at https://explore.plos.org/phishing.

If we can help with anything else, please email us at customercare@plos.org.

Thank you for submitting your work to PLOS One and supporting open access.

Kind regards,

PLOS One Editorial Office Staff

on behalf of

Dr. Tanay Chaubal

Academic Editor

PLOS One
